# Genome-wide identification of *TPS* and *TPP* genes in cultivated peanut (*Arachis hypogaea*) and functional characterization of *AhTPS9* in response to cold stress

**DOI:** 10.3389/fpls.2023.1343402

**Published:** 2024-01-19

**Authors:** Chao Zhong, Zehua He, Yu Liu, Zhao Li, Xiaoguang Wang, Chunji Jiang, Shuli Kang, Xibo Liu, Shuli Zhao, Jing Wang, He Zhang, Xinhua Zhao, Haiqiu Yu

**Affiliations:** ^1^ College of Agronomy, Shenyang Agricultural University, Shenyang, China; ^2^ Liaoning Agricultural Vocational and Technical College, Yingkou, China

**Keywords:** *TPS* genes, *TPP* genes, peanut (*Arachis hypogaea*), cold stress, trehalose

## Abstract

**Introduction:**

Trehalose is vital for plant metabolism, growth, and stress resilience, relying on *Trehalose-6-phosphate synthase* (*TPS*) and *Trehalose-6-phosphate phosphatase* (*TPP*) genes. Research on these genes in cultivated peanuts (*Arachis hypogaea*) is limited.

**Methods:**

This study employed bioinformatics to identify and analyze *AhTPS* and *AhTPP* genes in cultivated peanuts, with subsequent experimental validation of *AhTPS9’s* role in cold tolerance.

**Results:**

In the cultivated peanut genome, a total of 16 *AhTPS* and 17 *AhTPP* genes were identified. *AhTPS* and *AhTPP* genes were observed in phylogenetic analysis, closely related to wild diploid peanuts, respectively. The evolutionary patterns of *AhTPS* and *AhTPP* genes were predominantly characterized by gene segmental duplication events and robust purifying selection. A variety of hormone-responsive and stress-related *cis*-elements were unveiled in our analysis of *cis*-regulatory elements. Distinct expression patterns of *AhTPS* and *AhTPP* genes across different peanut tissues, developmental stages, and treatments were revealed, suggesting potential roles in growth, development, and stress responses. Under low-temperature stress, qPCR results showcased upregulation in *AhTPS* genes (*AhTPS2-5*, *AhTPS9-12*, *AhTPS14*, *AhTPS15*) and *AhTPP* genes (*AhTPP1*, *AhTPP6*, *AhTPP11*, *AhTPP13*). Furthermore, *AhTPS9*, exhibiting the most significant expression difference under cold stress, was obviously induced by cold stress in cultivated peanut, and *AhTPS9*-overexpression improved the cold tolerance of *Arabidopsis* by protect the photosynthetic system of plants, and regulates sugar-related metabolites and genes.

**Discussion:**

This comprehensive study lays the groundwork for understanding the roles of *AhTPS* and *AhTPP* gene families in trehalose regulation within cultivated peanuts and provides valuable insights into the mechanisms related to cold stress tolerance.

## 1 Introduction

Trehalose, a symmetrical non-reducing disaccharide formed by two glucose molecules through α, α-1,1-glycosidic bonds, exhibits distinctive physicochemical properties setting it apart from analogous sugars (Paul et al., 2008). It holds a pivotal role in maintaining cellular integrity across various organisms, particularly in plant growth and development (O’Hara et al., 2013). Under adverse conditions like high temperatures, freezing, and osmotic stress, cells synthesize trehalose as a defense mechanism, aiding in osmotic regulation, membrane preservation, and signal transduction (Fernandez et al., 2010; Kosar et al., 2019; Hassan et al., 2023). The precursor of trehalose is trehalose 6-phosphate (Tre6P), which has a dual role in plant metabolism and development (Figueroa and Lunn, 2016). It regulates sucrose production in source leaves to balance the demand for sucrose in growing sink organs. Tre6P also functions as a signal of sucrose availability, influencing developmental decisions such as flowering, embryogenesis, and lateral branching, thereby linking sucrose supply to sink organ growth (Paul et al., 2018). For example, Tre6P modulates plant respiration and metabolism by inhibiting SnRK1 activity, which functions as a key regulator of energy and nutrient responses in plants (Emanuelle et al., 2016). Trehalose synthesis, its versatile functions, and its presence in diverse organisms underline its significance in biological systems. This dynamic sugar, balancing energy and protective roles, holds promise for various applications in plant biotechnology and stress management.

Trehalose synthesis mainly involves two indispensable enzymes: trehalose 6-phosphate (Tre6P) synthase (TPS) and Tre6P phosphatase (TPP) (Smeekens, 2015). In plants, the trehalose biosynthesis pathway involves a sequential enzymatic process: Initially, trehalose-6-phosphate synthase (TPS) facilitates the condensation of uridine diphosphate glucose (UDPG) and glucose 6-phosphate (G6P) to yield an intermediary compound trehalose 6-phosphate (Tre6P). Subsequently, this intermediate is subjected to dephosphorylation by trehalose-6-phosphate phosphatase (TPP), resulting in the synthesis of trehalose (Fichtner and Lunn, 2021). TPS and TPP are key enzymes in the trehalose metabolism pathway. *TPS* and *TPP* genes are found in unicellular *chlorophyte algae*, *streptophyte algae*, and across all major groups of terrestrial plants, suggesting their presence from the early stages of the green plant lineage. Initially identified in *Arabidopsis* (*AtTPS1*, *AtTPPA*, and *AtTPPB*), these genes were subsequently recognized as part of extensive gene families after the sequencing of the *Arabidopsis* genome (Kaul et al., 2000). The *Arabidopsis* species possesses a total of 11 *TPS* genes (*AtTPS1*–*AtTPS11*) and 10 *TPP* genes (*AtTPPA*–*AtTPPJ*) (Aubourg et al., 2002; Vandesteene et al., 2012). The AtTPS proteins contain TPS and TPP domains, which form two distinct clades in phylogenetic analyses: class I (*AtTPS1*–*AtTPS4*) and class II (*AtTPS5*–*AtTPS11*) (Leyman et al., 2001). The *TPS* genes have been extensively studied in the model plant *Arabidopsis*, as well as in other plant species. These genes play a crucial role in serving as a conduit for sucrose signaling, triggering the production of trehalose during stress responses. The research conducted so far has illuminated the significance of *TPS* genes, particularly in terms of their capacity to enhance biotic and abiotic stress tolerance when overexpressed or properly expressed in transgenic plants (Iordachescu and Imai, 2008; Vishal et al., 2019; Fichtner et al., 2020). Furthermore, within plants, a substantial family of compact proteins housing the conserved only TPP domain is prevalent. In contrast, the functional roles of TPPs in the context of trehalose production have unveiled an array of distinct expression patterns across various tissues, underscoring their potential functional diversity (Vandesteene et al., 2012; Van Houtte et al., 2013; Du et al., 2022). It’s worth noting, however, that there does exist a degree of overlap in their expression patterns, suggesting a partial redundancy in their functions. Interestingly, through phylogenetic analysis, it has been possible to cluster each *TPP* and its counterparts from different species into distinct groups (Ma et al., 2007; Li et al., 2008). The induction of *TPP* expression comes about through varied hormonal and abiotic stress treatments, and the corresponding *TPPs* have been found to actively participate in plant stress responses. *AtTPPF*, for instance, assumes a proactive role in safeguarding cells against oxidative damage caused by reactive oxygen species during drought stress by augmenting soluble sugar levels (Lin et al., 2019). On the other hand, lines overexpressing *AtTPPD* display heightened salt tolerance due to increased sensitivity to redox shifts in two cysteine residues of *TPPD*. This sensitivity prompts accelerated activity under salt stress, thereby leading to an accumulation of trehalose (Krasensky et al., 2014). The overexpression of *OsTPS1* and *OsTPP1* within rice plants has been shown to bolster trehalose levels, ultimately enhancing plant survival under low temperature stress (Ge et al., 2008). Moreover, the latter protein, OsTPP1, contributes to the regulation of rice seed germination by engaging in ABA signaling (Wang et al., 2021). Both *TPS* and *TPP* gene families have independently expanded in various plant divisions, contributing to the diversity of stress response functions in plants.

Cultivated peanut (*Arachis hypogaea* L.) is an important oil and economic crop, which is widely planted in the semiarid tropical and subtropical regions (Krishna et al., 2015; Zhuang et al., 2019). Peanut plants are susceptible to various abiotic stresses during their growth, such as temperature, drought, salinity, and metal toxicit (Paul et al., 2001; Shi and Cai, 2009; Puppala et al., 2023). However, among various abiotic stresses, cold stress significantly impairs the growth, production, and quality of many crop plants (Kidokoro et al., 2022; Raza et al., 2023). In recent years, China has experienced an increased frequency of cold damage events, particularly impacting early-sown spring peanuts. These events have a common detrimental effect on various stages of peanut cultivation, including seed germination, growth, development, flowering, and overall yield (Kakani et al., 2002; Chen et al., 2014; Zhang et al., 2019; Xue et al., 2023; Zhang et al., 2023). Identifying key genes that can confer cold stress tolerance is crucial for enhancing crop productivity in regions with low temperatures (Bhat et al., 2022). These genes can be utilized in biotechnological programs to generate improved varieties, which is an urgent requirement in peanut production. Although the *TPS* and *TPP* genes has been identified to participate in growth, development, and response to various stress in multiple plant species, there have been no comprehensive analyses of the *TPS* and *TPP* gene families in peanut, and previous studies have not yet characterized the role of *TPS* and *TPP* genes in responding to environmental stresses. As a tetraploid crop, cultivated peanut contains A and B subgenomes that evolved from two diploid wild peanut varients (*Arachis duranensis* (AA) and *Arachis ipaensis* (BB). The sequencing of the whole genome of cultivated and wild peanut has been completed and uploaded, providing an opportunity for the analysis of the *TPS* and *TPP* gene familes in the context of cold stress in peanut (Bertioli et al., 2016; Bertioli et al., 2019; Chen et al., 2019; Zhuang et al., 2019).

In this study, we identified 16 *TPS* and 17 *TPP* genes in cultivated peanut, and comprehensively analyzed phylogenetic relationships, the chromosomal distributions, gene duplication, motif compositions, gene structures, and *cis*-acting elements of *TPS* and *TPP* genes using bioinformatics methods. Further transcriptome-based tissue-specific expression analysis of *AhTPS* and *AhTPP* genes was conducted, and their expression patterns in response to low temperature were assessed using fluorescence quantitative PCR (qPCR) methods. Among these, key genes were functionally characterized in transgenic *Arabidopsis* to reveal its crucial role in cold tolerance. Our findings lay the groundwork for further investigation into the functions of the *AhTPS* and *AhTPP* families in cultivated peanut. Additionally, this research will facilitate their utilization in the genetic improvement of crops.

## 2 Materials and methods

### 2.1 Plant materials and treatment

The cold-stress variety Nonghua5 provided by the Peanut Research Institute, Shenyang Agriculture University, Shenyang, China, was used for cultivation and cold treatment. The peanuts underwent sowing, cultivation, and cold treatment following the protocol outlined in Zhang et al. (2023), with minor adjustments. To ensure sterility, the seeds were surface treated using a 3% sodium hypochlorite solution, rinsed thoroughly with distilled water, and placed in darkness for germination. Germinated seeds were then planted in circular plastic pots filled with a mixture of vermiculite and nutrient soil in a 2:1 ratio. The plants were grown in a climate chamber with a 16-hour light (28°C)/8-hour dark (23°C) cycle, a photosynthetic photon flux density of 400 μmol m^−2^ s^−1^, and a relative humidity of 70%. To induce cold stress, the temperature in the climate chamber was lowered to 4°C while maintaining other growth conditions. The second leaves were collected at 0, 6, 12, 24, and 48 hours after each treatment, with three biological replicates. The collected leaves were immediately frozen in liquid nitrogen and stored at -80°C.

### 2.2 Identification of the *TPS* and *TPP* family genes in cultivated peanut and wild peanut

To identify genes encoding TPS and TPP proteins of tetraploid cultivated peanuts (*Arachis hypogaea*) and diploid wild peanuts (*Arachis duranensis* and *Arachis ipaensis*) in Peanut genome database (PeanutBase-BLAST, https://www.peanutbase.org/pb_sequenceserver), the sequences of 11 AtTPS and 10 AtTPP proteins were utilized, with an E-value threshold of < 10^-5^. The identified *TPS* and *TPP* gene sequences underwent a conserved domain search (http://www.ncbi.nlm.nih.gov/Structure/cdd/wrpsb.cgi), and manually removing peanut protein sequences lacking TPS or TPP domains. SMART (http://smart.embl.de/) and Pfam (http://pfam.xfam.org/) were employed to confirm the conserved domains of TPS and TPP using the remaining protein sequences. The ExPASy database (https://web.expasy.org/protparam/) was utilized to predict the physicochemical properties of *AhTPSs* and *AhTPPs*, including molecular weight, isoelectric point (pI), aliphatic index, and total average hydrophilicity (GRAVY).

### 2.3 Phylogenetic analysis of *AhTPS* and *AhTPP* genes

The phylogenetic tree was constructed using protein sequences from *TPS* and *TPP* genes in cultivated and wild diploid peanuts (*Arachis hypogae*a, *Arachis duranensis*, and *Arachis ipaensis*), as well as *Arabidopsis*, soybean, rice, and tomato. The protein sequences of TPP and TPS from *Arachis hypogaea*, *Arachis duranensis*, and *Arachis ipaensis* were obtained from the peanut genome database (https://www.peanutbase.org/). Similarly, the protein sequences of TPP and TPS from *Arabidopsis* and rice were downloaded from NCBI (https://www.ncbi.nlm.nih.gov/), while those from soybean and tomato were obtained from the soybean genome database (https://soybase.org/). Multiple sequence alignments were performed using the MUSCLE method, and the phylogenetic tree was constructed using the neighbor-joining (NJ) method with 1000 bootstrap replications, employing MEGA11 software (Tamura et al., 2021). The resulting tree was visualized using EvolView (https://evolgenius.info//evolview).

### 2.4 Chromosomal location, gene duplication, and synteny analysis

The chromosomal distribution of *AhTPSs* and *AhTPPs* was determined by MG2C online software. Gene duplication patterns and collinearity relationships of the *AhTPS* and *AhTPP* gene families between tetraploid cultivated and diploid wild peanuts were identified and analyzed using MCScanx of TBtools with default parameters. TBtools was subsequently used to calculate Ka and Ks, with a Ka/Ks to explore the evolutionary dynamics and selection pressure (Chen et al., 2020).

### 2.5 Analysis of gene structures and motifs

Based on the peanut genome annotation (GFF file), gene exon-intron structures were obtained. TBtools was utilized to construct protein motif and gene structure maps for members of the peanut *TPS* and *TPP* gene families. The MEME program (http://meme-suite.org/tools/meme) was employed to assay the conserved motifs of each protein. The parameters used to identify conserved motifs in the protein sequences were set as follows: a maximum of 20 motifs and other optional default parameters.

### 2.6 Cis-acting element prediction in the upstream region of *TPPs* and *TPSs*

In order to explore potential *cis*-acting regulatory elements within the promoter regions of *AhTPP* and *AhTPS* genes, the upstream sequence of 2000 bp preceding the coding region was extracted from the peanut genome sequence. Subsequently, this sequence was subjected to *cis*-regulatory element prediction using the PlantCARE database (https://bioinformatics.psb.ugent.be/webtools/plantcare/html/). The predicted *cis*-regulatory elements were categorized based on their regulatory functions, and their distribution within the promoter regions of *AhTPP* and *AhTPS* was visualized as a heatmap.

### 2.7 Expression profile analysis of *AhTPSs* and *AhTPPs* in different tissues and under different environmental treatments

The transcriptome-based data of different tissues and under different environmental treatments (different hormonal, low temperature and drought treatments) were retrieved from the PeanutBase database (https://www.peanutbase.org/) and Peanut Genome Resource (http://peanutgr.fafu.edu.cn/index.php) (Clevenger et al., 2016; Zhuang et al., 2019). TBtools was used to generate an expression heatmap of the reads per kilobase per million mapped reads (RPKM) data. The transcriptome data were normalized by log_2_ (1 +RPKM).

### 2.8 RNA extraction and quantitative real-time PCR

Total RNA of plant tissues was isolated using the Plant Total RNA Extraction Kit (Tiangen Biotech, Beijing, China) according to the manufacturer’s instructions. Then, cDNA was synthesized by PrimeScriptTM RT Kit (TaKaRa, Japan). *AhActin* was used as the reference gene, and the specific primers are designed using Primer-BLAST (https://www.ncbi.nlm.nih.gov/tools/primer-blast/). The gene expression analysis was carried out using the SYBR Premix Ex TaqII kit (TliRNaseH Plus) from TaKaRa, Japan, and fluorescence quantitative reactions were detected using ABI7500 from Applied Biosystems, United States. The relative expression analysis was calculated using the 2^−ΔΔCT^ approach.

### 2.9 Subcelluar localization analysis of AhTPS9 protein

The specific primers (*AhTPS9*-F/R) were utilized to amplify the complete cDNA of *AhTPS9* from tobacco leaves. The coding sequence data for *AhTPS9* from PeanutBase (https://www.peanutbase.org/) were used as a reference. The resulting PCR products were connected to the pBWA (V) HS-GLosgfp vector, generating the pBWA (V) HS- *AhTPS9-*GFP vector which incorporates the green fluorescent protein (GFP) reporter gene. After confirmation through sequencing, the positive clones were transferred into *Agrobacterium tumefaciens* (EHA105) using electrotransformation. Young seedlings (30 days old) were carefully chosen, and injections were performed into the lower epidermis of leaves. Subsequently, these seedlings were cultured under low light conditions for 2 days. Observation and imaging took place using a laser confocal microscope (Nikon C2-ER, Tokyo, Japan), with the corresponding empty vector serving as a control.

### 2.10 The *AhTPS9* function analysis under cold stress conditions

Gene-specific primers for *AhTPS9* were designed to amplify the cDNA sequence in a cold-tolerance genotype Nonghua5 by Primer Premier 6.0. PCR amplification of the coding sequence of *AhTPS9* was performed using the TransTaq DNA Polymerase High Fidelity Amplification Kit. The PCR reaction program consisted of an initial denaturation at 94°C for 3 minutes, followed by 30 cycles of denaturation at 94°C for 30 seconds, annealing at 58-60°C for 30 seconds, and extension at 72°C for 1 minute. The final extension was carried out at 72°C for 10 minutes. PCR products were analyzed on a 1% agarose gel, purified using a universal DNA purification recovery kit, and then ligated into the pBWA (V) BS cloning vector. The ligated DNA was transformed into *Escherichia coli Top10* competent cells, positive clones were selected, and their identity was confirmed through sequencing. The recombinant plasmid pBWA (V) BS-*AhTPS9* was transformed into *Agrobacterium tumefaciens* EHA105, which was subsequently introduced into wild-type (WT) *Arabidopsis* using the floral dip method as described by Clough and Bent (1998). Following antibiotic-based screening and PCR verification of the transgenic seedlings, we successfully generated the homozygous transgenic lines in the T_2_ generation. Subsequently, homozygous T_3_ progeny was examined and selected for further experimental procedures.

The T_3_ seeds, previously subjected to a 4°C vernalization treatment on MS medium, were incubated in a growth chamber at 22°C for 14 days. Subsequently, they underwent a 5-day cold treatment at 4°C, followed by phenotypic observations. Columbia wild-type *Arabidopsis* plants (WT) were subjected to the same cold treatment conditions as controls. Leaf samples were collected at 0h (CK) and 24h after the cold treatment to assess changes in the expression levels of low-temperature-responsive genes in both overexpressing plants and wild-type plants. At 72h after the low-temperature treatment, leaf samples were collected to analyze related metabolites and physiological indicators. All samples are stored in liquid nitrogen for further use.

### 2.11 Physiological indicator and metabolism measurements

To evaluate the physiological indicators in transgenic and wild plants under cold conditions, the contents of proline and MDA, soluble sugar and starch content were measured using the commercial kits according to the manufacturer’s instructions (Solarbio, Beijing, China). To determine chlorophyll content, leaves of wild-type and transgenic *Arabidopsis* plants, with and without cold stress treatment, were homogenized in 80% acetone. The homogenized leaves were refrigerated at 4°C for 3 days until they reached near-bleached status. Optical density (OD) values at 645nm and 663nm were then measured, and chlorophyll content was calculated using the formula: (8.02*OD663 + 20.21*OD645) *M/1000/N, where M represents the volume of 80% acetone added, and N is the fresh weight of the aerial plant parts. “The net photosynthetic rate, Fv/Fm, and electrolytic leakage photosynthetic indices were measured using the Li-6400 portable photosynthetic apparatus. Fv/Fm measurements were conducted using the Dual Pam 100, with *Arabidopsis* plants darkened for a minimum of 30 minutes prior to measurement. The electrolytic leakage experiment method was referenced from Bajji et al. (2002). The measurement of sugar metabolism products, including trehalose, Tre6p, and sucrose content, was conducted using high-performance liquid chromatography-mass spectrometry (HPLC-MS), following the method outlined in Lin et al. (2023).

### 2.12 Statistical analysis

Three replicates were used for all experiments. Statistically significant data were analyzed using one-way analysis of variance (ANOVA) with the least significant difference (LSD) method. The R package ANOVA (https://statsandr.com/blog/anova-in-r/) was employed for this analysis. The calculated values were presented as means ± standard deviation (SD).

## 3 Result

### 3.1 Genome-wide identification and characteristics of *AhTPS* and *AhTPP* genes in wild and cultivated peanut

Through BLAST searches and confirmation of conservative domains, a total of 16 *TPS* and 17 *TPP* genes were identified in the cultivated peanut genome (*Arachis hypogaea*). Based on their chromosomal locations, these *TPS* genes were designated as *AhTPS1-16*, while the *TPP* genes were named *AhTPP1-17*. All 16 *TPS* genes contained both *TPS* and *TPP* conserved domains, whereas the 17 *TPP* genes each contained a single typical *TPP* domain (**Supplementary Figures 1**, **2**). Additionally, 19 *TPS* genes and 15 *TPP* genes were identified in the wild diploid peanut genomes *Arachis duranensis* and *Arachis ipaensis*. In the *A. duranensis* genome, 9 *TPS* genes and 7 *TPP* genes were identified and named *AdTPS1* – *AdTPS9* and *AdTPP1* – *AdTPP7*, respectively. Meanwhile, *A. ipaensis* harbored 10 *TPS* and 8 *TPP* genes designated as *AiTPS1* – *AiTPS10* and *AiTPP1* – *AiTPP8* (**Tables 1**, **2**).

**Table 1:** Characteristics of *TPS* family members in cultivated peanut (*Arachis hypogaea*).

Gene name	mRNA ID	Genome location	Number of amino acids	Length of gene/bp	Molecular weight/Da	Isoelectric point (pI)	GRAVY	Aliphatic index
***AhTPS1***	*Arahy.93VR75*	Chr01:87159326-87162241	705	848	80392	6.89	-0.283	85.02
***AhTPS2***	*Arahy.G88L7W*	Chr01:96802522-96809236	849	916	96026.65	5.81	-0.226	87.68
***AhTPS3***	*Arahy.RIH8EG*	Chr03:24399601-24405854	1003	973	113229.45	6.22	-0.275	86.12
***AhTPS4***	*Arahy.IE8W25*	Chr03:133371847-133376625	862	862	97108.04	5.88	-0.189	91.58
***AhTPS5***	*Arahy.Q3GMD8*	Chr03:136252971-136257365	854	1003	96681.96	5.77	-0.188	91.63
***AhTPS6***	*Arahy.E48PAY*	Chr07:76447357-76459343	927	1022	104664.21	7.01	-0.383	84.03
***AhTPS7***	*Arahy.BL0RCS*	Chr09:113085191-113089493	847	853	95865.65	5.6	-0.216	88.58
***AhTPS8***	*Arahy.A0DAR9*	Chr11:112046409-112051186	848	829	96610.43	5.91	-0.221	86.99
***AhTPS9***	*Arahy.1XW75L*	Chr11:147434575-147439870	849	849	95996.62	5.81	-0.225	87.68
***AhTPS10***	*Arahy.FTDL1P*	Chr13:26117125-26123363	973	849	109322.73	5.81	-0.26	84.76
***AhTPS11***	*Arahy.CM68RF*	Chr13:135800911-135805745	862	860	97165.09	5.79	-0.184	91.8
***AhTPS12***	*Arahy.FP7P7G*	Chr13:138499685-138504202	853	854	96618.88	5.77	-0.195	91.62
***AhTPS13***	*Arahy.PK6QLT*	Chr13:145969353-145977152	1022	174	116170.19	8.31	-0.296	89.18
***AhTPS14***	*Arahy.AKPI0I*	Chr15:26507553-26513085	860	855	96986.1	6.11	-0.175	93
***AhTPS15***	*Arahy.FZMU71*	Chr15:130260497-130267100	869	862	98141.75	5.73	-0.225	86.94
***AhTPS16***	*Arahy.9VZ5EJ*	Chr19:156034428-156039218	855	847	96691.62	5.69	-0.196	89.58

**Table 2:** Characteristics of *TPP* family members in cultivated peanut (*Arachis hypogaea*).

Gene name	mRNA ID	Genome location	Number of amino acids	Length of gene/bp	Molecular weight/Da	Isoelectric point (pI)	GRAVY	Aliphatic index
*AhTPP1*	*Arahy.FIK9JS*	Chr03:10868380-10873244	388	388	43213.37	6.57	-0.275	88.92
*AhTPP2*	*Arahy.P5P8C7*	Chr03:124464638-124467355	355	391	39933.8	9.08	-0.392	80.76
*AhTPP3*	*Arahy.51Y4LP*	Chr05:14048218-14053780	386	279	43296.31	6.22	-0.381	85.83
*AhTPP4*	*Arahy.J3QXZL*	Chr07:57504749-57508748	312	388	35412.85	9.31	-0.366	88.4
*AhTPP5*	*Arahy.0FY2NM*	Chr08:8204445-8216930	373	373	42405.02	9.22	-0.332	85.2
*AhTPP6*	*Arahy.B1753N*	Chr08:12717424-12719898	309	327	34895.17	9.07	-0.348	87.73
*AhTPP7*	*Arahy.B2J19D*	Chr08:49036435-49039546	279	245	31689.36	7.79	-0.295	92.22
*AhTPP8*	*Arahy.SS1VDK*	Chr10:12843901-12849157	391	355	43915.47	9.04	-0.353	81.99
*AhTPP9*	*Arahy.U769C2*	Chr12:4517116-4529201	334	378	38130.21	5.49	-0.375	86.65
*AhTPP10*	*Arahy.8AAC5A*	Chr12:27460283-27462833	245	129	27864.94	5.44	-0.203	85.06
*AhTPP11*	*Arahy.CK7SVG*	Chr13:14488386-14492535	445	437	49874.19	7.59	-0.256	88.49
*AhTPP12*	*Arahy.FJ2B1F*	Chr13:127868945-127871708	355	374	39946.89	9.22	-0.393	80.76
*AhTPP13*	*Arahy.5Q8BGE*	Chr15:14981830-14987321	375	386	42051.76	6.03	-0.41	86.27
*AhTPP14*	*Arahy.DP0G5T*	Chr17:56617699-56620895	313	335	35206.75	9.48	-0.296	83.13
*AhTPP15*	*Arahy.G2WE73*	Chr17:122383126-122395867	373	334	42431.04	9.33	-0.343	85.2
*AhTPP16*	*Arahy.KW1U5A*	Chr17:128686117-128688689	327	324	36737.21	9.18	-0.378	85.87
*AhTPP17*	*Arahy.S15BQI*	Chr20:20822125-20826715	374	313	41950.96	9.12	-0.472	74.25

Physicochemical property analysis revealed that the gene length of *AhTPSs* ranged from 174 to 1022 bp, protein length from 705 to 1022 amino acids, molecular weight from 80.4 kDa to 116.17 kDa. The aliphatic index of *AhTPSs* ranged from 84.03 to 91.58. Isoelectric points of *AhTPS1*-*AhTPS5*, *AhTPS7*-*AhTPS12*, and *AhTPS14*-*AhTPS16* fell within the range of 5.01 to 6.89, indicating an acidic nature due to an abundance of acidic amino acids. In contrast, *AhTPS6* and *AhTPS13* were enriched in basic amino acids. Gene length for *AhTPPs* ranged from 129 to 437 bp, protein length from 245 to 445 amino acids, and molecular weight from 27864.94 Da to 49874.19 Da. The aliphatic index of *AhTPPs* ranged from 74.25 to 92.22. Theoretical isoelectric point analysis indicated that *AhTPP1, AhTPP3, AhTPP9*, *AhTPP10* and *AhTPP13* had isoelectric points between 5.01 and 6.57, suggesting an acidic nature due to acidic amino acids. Conversely, *AhTPP2, AhTPP4*-*AhTPP8*, *AhTPP11*-*AhTPP12*, and *AhTPS14*-*AhTPS17* were rich in basic amino acids. The GRAVY (Grand Average of Hydropathy) values for all 16 TPS and 17 TPP proteins were below 0, indicating their hydrophilic nature.

### 3.2 Phylogenetic analysis of *AhTPS* and *AhTPP* gene families

**Figure 1:**
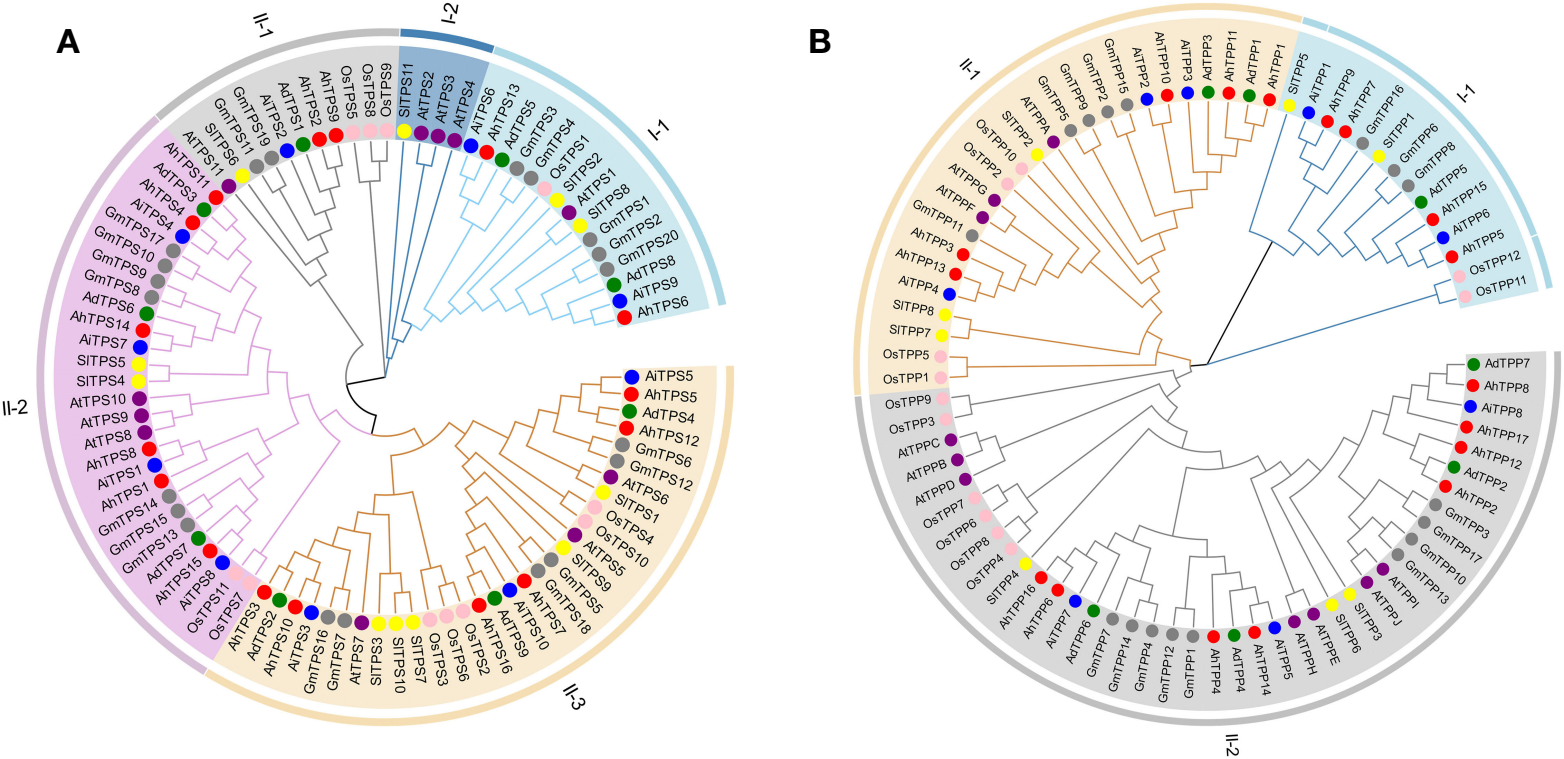
Phylogenetic analysis of *TPS* **(A)** and *TPP* **(B)** gene family among cultivated peanut (*Arachis hypogaea*, *Ah*), wild diploid peanut (*Arachis duranensis*, *Ad and Arachis ipaensis*, *Ai*), *Arabidopsis thaliana* (*At*), soybean (*Glycine max*, *Gm*), rice *(Oryza sativa*, *Os*) and tomato (*Solanum lycopersicum*, *Sl*). The phylogenetic tree was constructed using MEGA11 based on the Neighbor-joining method with 1000 bootstrap replicates.

To investigate the evolutionary relationships of the *AhTPS* and *AhTPP* gene families, phylogenetic trees were constructed using *TPS* and *TPP* family genes from *Arabidopsis*, soybean, rice, tomato, wild diploid peanut, and cultivated peanut, respectively. Across these species, a total of 87 *TPS* genes and 79 *TPP* genes were used to construct systematic phylogenetic trees. In these species, a total of 87 *TPS* genes and 79 *TPP* genes were employed to build systematic phylogenetic trees. Based on the phylogenetic relationship of rice and *Arabidopsis* (**Figure 1A**), The *TPS* gene family is categorized into two subfamilies, namely Class I and Class II. Within Class I, there are two subgroups, while Class II is divided into three subgroups. *AtTPS1-4* belong to Class II, while *AtTPS5-11* belong to Class I. The gene counts of *TPS* within different taxa, including cultivated peanut, two wild diploid peanuts (Ad/Ai), rice, soybean, *Arabidopsis*, and tomato, are as follows: Class I-1 (2, 2/2, 1, 5, 1, 2), Class I-2 (0, 0/0, 0, 0, 3, 1), Class II-1 (2, 1/1, 3, 2, 1, 1), Class II-2 (6, 3/4, 2, 7, 3, 2), and Class II-3 (6, 3/3, 5, 6, 3, 5). Except for Class I-2, each group contains at least one gene across the seven species. Comparative analysis suggests a closer genetic relationship between *TPS* genes in the *Arachis* genus and *GmTPS* genes in soybean. Furthermore, *AhTPS6* in Class I-1 clusters alongside *AtTPS1* and *OsTPS1*, implying potential functional similarity among genes within the same group.

The *TPP* gene family is divided into two categories (**Figure 1B**). Class I *TPP* genes consist of four cultivated peanut genes (*AhTPP5*, *AhTPP7*, *AhTPP9*, and *AhTPP15*), three wild peanut genes (*AdTPP5*, *AiTPP1*, and *AiTPP6*), three soybean genes (*GmTPP6*, *GmTPP8*, and *GmTPP16*), two rice genes (*OsTPP11* and *OsTPP12*), and two tomato genes (*SlTPP1* and *SlTPP5*); the remaining thirteen *TPP* genes belong to Class II. Class II *TPP* genes are divided into two subgroups. *AtTPPA*, *AtTPPG*, and *AtTPPF* belong to Class II-1, while the rest of the *AtTPP* genes belong to Class II-2. Gene counts within groups for cultivated peanut, wild peanut (Ad/Ai), rice, soybean, Arabidopsis, and tomato are as follows: Class I-1 (4, 1/2, 2, 3, 0, and 2), Class II-1 (5, 2/3, 4, 5, 3, and 3), and Class II-2 (8, 4/3, 6, 9, 7, and 3). The phylogenetic relationships between *AhTPPs*, *GmTPPs*, and *AtTPPs* are closer. *AhTPP1*, *AhTPP3*, *AhTPP10*, *AhTPP11*, along with *AtTPPA*, *AtTPPG*, and *AtTPPF*, cluster in Class II-1. *AhTPP6* and *AhTPP16*, along with *AhTPP4*, *AhTPP14*, *AhTPP2*, *AhTPP12*, *AhTPP17*, and *AhTPP8*, cluster in Class II-2. *AtTPPB*, *AtTPPC*, *AtTPPD*, *AtTPPE*, *AtTPPH*, *AtTPPI*, and *AtTPPJ* also cluster in Class II-2. Altogether, *TPS* and *TPP* genes exhibit close relationships within subgenomes of cultivated peanuts and wild diploid peanuts, and share a higher degree of similarity with leguminous crop, soybean.

### 3.3 Chromosomal location, gene duplication, and synteny analysis of *AhTPS* and *AhTPP* genes

**Figure 2:**
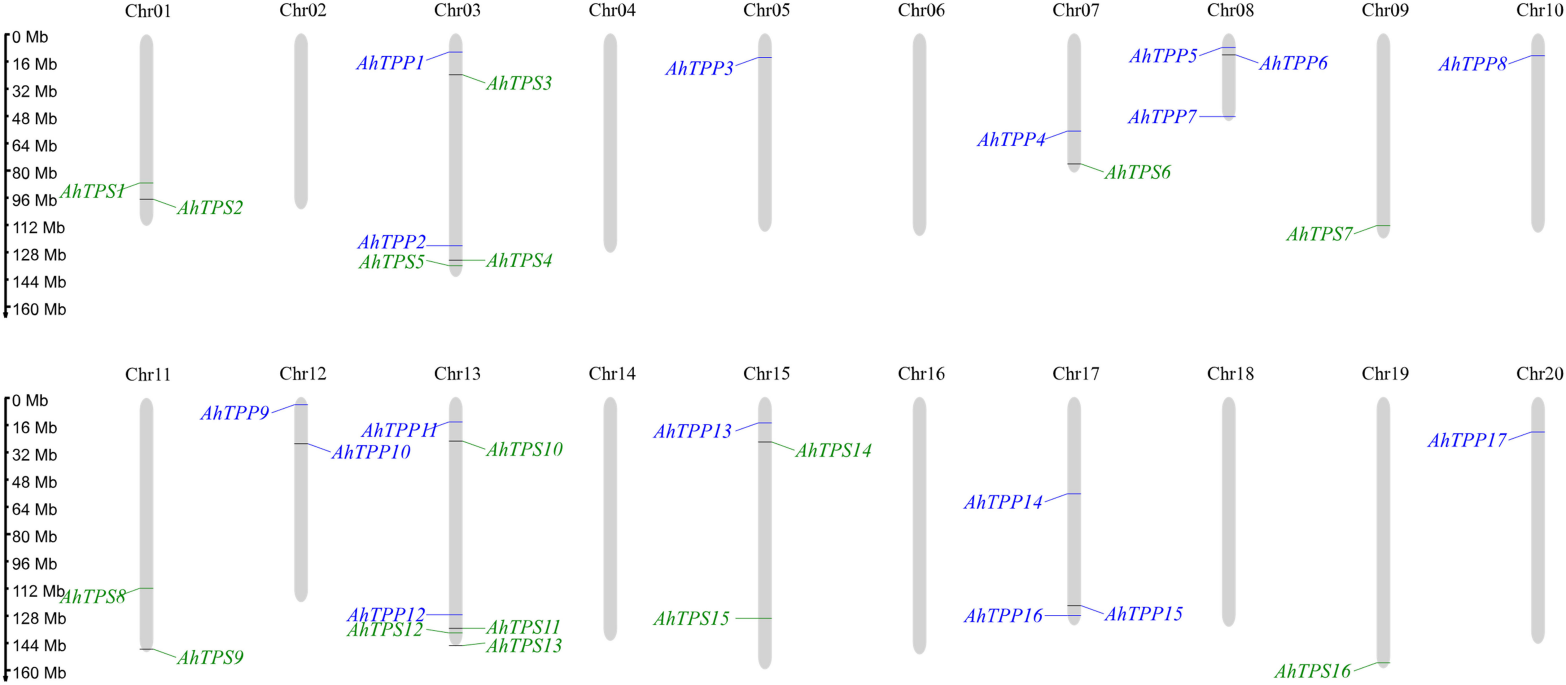
Chromosomal location analysis of *AhTPS* and *AhTPP* genes. *AhTPSs* (green) and *AhTPPs* (blue) are marked on chromosomes. The scale bar on the left indicates the length of *A. hypogaea* chromosomes (Mb).

To study the chromosomal locations of *AhTPS* and *AhTPP* genes in the genome of cultivated peanut, the distribution of genes on chromosomes is depicted based on the genome annotation file (GFF) of cultivated peanut (**Figure 2**). We observed an uneven distribution of the *AhTPS* and *AhTPP* genes on the chromosomes of cultivated peanut. The results indicate that peanuts have 20 chromosomes, with 16 *AhTPS* genes distributed across Chrs01, 03, 07, 09, 11, 13, 15, and 19. Seventeen *AhTPP* genes are distributed across chromosomes 03, 05, 07, 08, 10, 12, 13, 15, 17, and 20. Most of these genes are located near the ends of the chromosomes. *TPS* and *TPP* genes were not identified on certain chromosomes, such as Chr02, Chr04, Chr06, Chr14, Chr16, and Chr18. Based on the gene locations on chromosomes, it was found that some *TPS* and *TPP* orthologous genes from the A subgenome (Chr01-10) and the B subgenome (Chr11-20) exhibit consistent positions, such as *AhTPS1* and *AhTPS8*, *AhTPS2* and *AhTPS9*, *AhTPP1* and *AhTPP11*. It suggests their retention during the evolution from wild diploid peanuts to cultivated tetraploid peanut. Conversely, certain genes show distribution differences between the A and B subgenomes. For instance, *TPP* genes on Chr02 have been lost during the evolutionary process, while Chr12 retains *AhTPP9* and *AhTPP10*.

**Figure 3:**
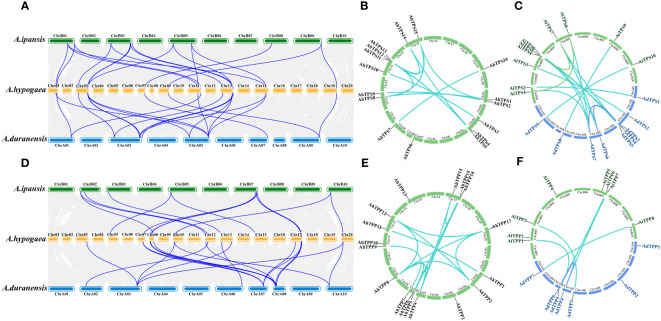
Collinearity analysis of the *TPS*s **(A–C)** and *TPP*s **(D–F)** in three *Arachis* species. **(A)** The syntenic relationship of the *AhTPSs* between *A. ipaensis, A. hypogaea* and *A. duranensis* genomes. **(B)** The collinearity relationship of *AhTPSs* within the cultivated peanut (*A. hypogaea*) genome. **(C)** The collinearity relationship of *AhTPSs* between wild diploild peanut *A. ipaensis* and *A. duranensis* genomes. **(D)** The syntenic relationship of the *AhTPPS*s between *A. ipaensis, A. hypogaea* and *A. duranensis* genomes. **(E)** The collinearity relationship of *AhTPPs* within the cultivated peanut (*A. hypogaea*) genome. **(F)** The collinearity relationship of *AhTPPs* between wild diploild peanut *A. ipaensis* and *A. duranensis* genomes.

To study evolutionary relationships and reveal the homology relationship of *TPS* and *TPP* genes in different *Arachis* species, two diploid wild peanut, *Arachis duranensis* and *Arachis ipaensis*, were selected for collinear analysis the cultivated peanut *Arachis hypogaea* (**Figure 3**). According to collinearity analysis, 22 and 21 collinear gene pairs were detected between *AhTPS* genes of *A. duranensis* and *A. hypogaea*, and *A. ipaensis* and *A. hypogaea*, respectively (**Figure 3A**, **Supplementary Table 1**). Between *AhTPP* genes of *A. duranensis* and *A. hypogaea*, as well as *A. ipaensis* and *A. hypogaea*, 19 and 14 collinear gene pairs were respectively identified (**Figure 3D**, **Supplementary Table 2**). All *TPS* and *TPP* members in cultivated peanuts possess corresponding segments in both wild species, suggesting the genetic conservation of these two family members during the formation of tetraploid peanuts from their wild counterparts. Gene duplication (segmental and tandem duplication) is a major force behind genome evolution. So, *Arachis TPS* and *TPP* gene duplication events were evaluated (**Supplementary Tables 3**, **4**). The *AhTPS* gene family exhibited 12 pairs of segment duplications (**Figure 3B**), while the *AhTPP* gene family showed 16 pairs of segment duplications (**Figure 3E**). No tandem duplications were identified in either the *AhTPS* or *AhTPP* gene families. Further collinearity analysis was conducted between the gene families in the diploid wild peanut genomes. Among the *TPS* gene families of the two wild species, 13 pairs of orthologous genes were identified (**Figure 3C**), and for the *TPP* gene families, 6 pairs of orthologous genes were detected (**Figure 3F**). Some gene pairs, such as *AdTPS3-AdTPS6*, *AdTPS3-AdTPS7*, and *AiTPS1-AiTPS8* in the *TPS* gene family (**Supplementary Table 3**), as well as *AdTPP4-AdTPP6* in the *TPP* gene family (**Supplementary Table 4**), suggested that these segment duplications had already were present during the evolution of wild diploid peanuts. Discrepancies between the collinearity maps of wild diploid and cultivated peanuts may be attributed to the emergence of new segment duplications or transpositions within the *TPS* and *TPP* gene families after the derivation of tetraploid cultivated peanuts from diploid wild peanuts. The ratio of nonsynonymous substitution rate (Ka) to synonymous substitution rate (Ks) provides insights into the evolutionary process and selection pressure. We calculated the Ka/Ks ratio among the *Arachis* species. For both *AhTPS* and *AhTPP* gene members, except for a few genes for which Ka/Ks ratios could not be calculated, most of the calculated Ka/Ks ratios were less than 1, indicating that *AhTPS* and *AhTPP* genes were subject to strong purifying selection pressure during evolution (**Supplementary Tables 5**, **6**).

### 3.4 Gene structure and motif composition of *AhTPS* and *AhTPP* genes

**Figure 4:**
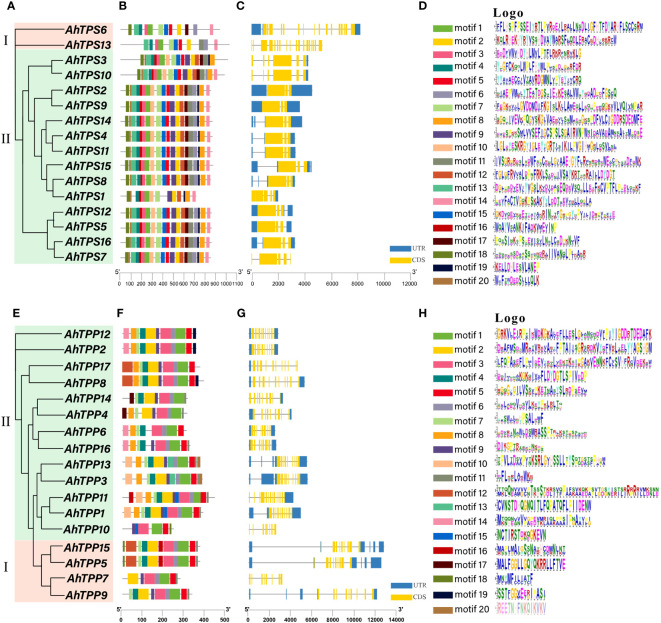
The phylogenetic trees, motifs, and gene intron/exon structures of 16 *AhTPS* genes and 17 *AhTPP* genes in cultivated peanut. **(A, E)** Phylogenetic tree based on AhTPS and AhTPP protein sequences using Neigbour-joining Tree method. **(B, F)** The motif compositions of AhTPS and AhTPP proteins. specific motifs were indicated using different colors. **(C, G)** The exon and intron distribution of *AhTPS* and *AhTPP* genes. Exons and intron regions are represented by yellow rectangles and grey lines, respectively. **(D, H)** Sequence logos of 20 motifs in cultivated AhTPS and AhTPP proteins, respectively.

Gene structure analysis played a crucial role in elucidating the connection between gene family evolution and functional divergence. To unveil the inherent structural attributes of the *AhTPS* and *AhTPP* families, an in-depth investigation of gene structures and conserved motifs was conducted (**Figure 4**). Based on the phylogenetic analysis (**Figure 1**; **Figures 4A,E**), Both *AhTPS* and *AhTPP* members were divided into 2 groups (I and II), respectively, and a total of 20 conserved motifs are identified (**Figures 4B,D**). In the *AhTPS* gene family, Class I genes possess a greater number of introns (19 and 17) compared to Class II genes (4, 5, or 6), indicating a more complex gene structure (**Figure 4C**). Genes with close phylogenetic relationships often exhibit higher structural similarity. Among the Class I *AhTPS* genes, *AhTPS3* and *AhTPS10* have 6 introns each, while *AhTPS2* and *AhTPS9* have 4 introns each. Concerning the *AhTPP* gene family, Class I *AhTPP* genes, *AhTPP5* and *AhTPP15*, have the highest number of introns at 14, whereas *AhTPP7* possesses only 7 introns (**Figure 4G**). In Class I *AhTPP* genes, *AhTPP11* has 14 introns, *AhTPP8* and *AhTPP17* have 11 introns, *AhTPP2* and *AhTPP12* contain 12 introns, *AhTPP6* and *AhTPP16* have 10 introns, and *AhTPP8* and *AhTPP17* possess 11 introns. Subsequently, we employed the online tool MEME to identify conserved motifs within AhTPS and AhTPP proteins. All AhTPS proteins contain motifs 1, 2, 3, 4, 5, 7, and 15. Except for AhTPS1, all AhTPS proteins contain motifs 6 and 13, and motifs 18 are present in all AhTPS proteins except AhTPS13. Most AhTPS proteins encompass motifs 8, 9, 10, 11, 12, 16, 17, 19, and 20. The fewest motifs (11) are found in AhTPS13. Generally, AhTPP proteins exhibit fewer motifs compared to AhTPS proteins, with AhTPP11 having the lowest count of motifs (13) (**Figures 4F,H**). All AhTPP proteins share motif 1, while motifs 13 and 20 are exclusively present in AhTPP3 and AhTPP13, motif 17 only in AhTPP4 and AhTPP14, and motif 18 only in AhTPP5 and AhTPP10. Identified conserved domains are listed according to their conservation levels within the *AhTPS* (**Figure 4D**) and *AhTPP* (**Figure 4H**) gene families. The results reveal that the most conserved motifs in *AhTPS* are generally located at the N-terminal, whereas the conserved motifs in the *AhTPP* family are predominantly situated at the C-terminal. This could be attributed to the positioning of TPS domains at the N-terminal and TPP domains at the C-terminal.

### 3.5 *Cis-*element analysis in the upstream region of *AhTPS* and *AhTPP* genes

**Figure 5:**
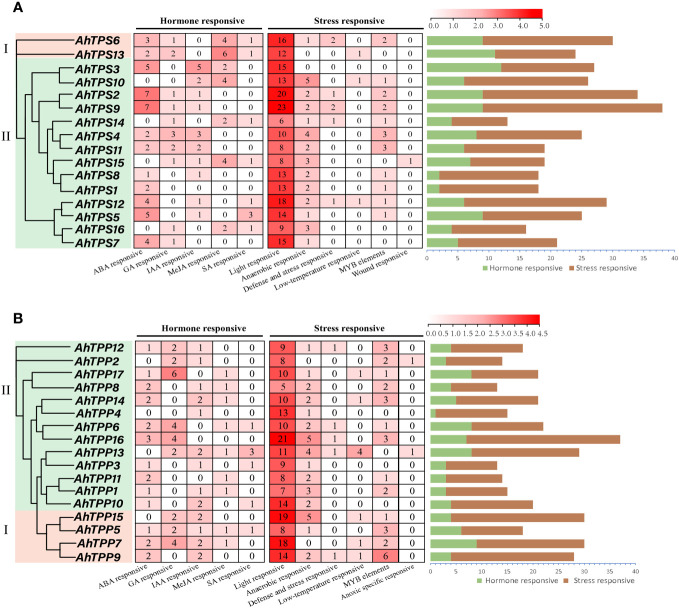
*Cis*-regulatory elements in the promoters of *AhTPS* **(A)** and *AhTPP* **(B)** genes in peanut. Various *cis*-regulatory elements are displayed in different colored boxes.

*Cis-*elements are necessary for gene expression and are widely involved in the regulation of plant growth and development and stress response. To further investigate the potential regulatory mechanisms of the *AhTPS* and *AhTPP* genes under stress conditions, we predicted and summarized the *cis*-regulatory elements within the first 2000 bp segments upstream of *AhTPSs* and *AhTPPs*, respectively (**Supplementary Tables 7**, **8**). Cluster analysis of *cis*-regulatory elements in promoter regions reveals that orthologous genes from A and B subgenomes of cultivated peanut exhibit similar *cis*-element distributions, both in *AhTPS* and *AhTPP* genes (**Figure 5**). In the *AhTPS* genes, a total of 61 *cis*-regulatory elements were identified, including hormone-responsive elements such as abscisic acid (ABA), auxin (IAA), gibberellin (GA), salicylic acid (SA), and methyl jasmonate (MeJA) response elements, as well as stress-related elements like light response, anaerobic response, low temperature, wound, and MYB elements. Almost all *AhTPS* genes contain a significant number of light-responsive elements. However, considerable variation exists among different genes with respect to other hormones and stress responses. For instance, *AhTPS2*, *AhTPS5*, *AhTPS9*, and *AhTPS12* contain numerous ABA-responsive elements, while *AhTPS6* and *AhTPS13* have a higher abundance of MeJA-responsive elements. In *AhTPP* genes, a total of 54 *cis*-regulatory elements were identified, including elements responsive to hormones such as ABA, MeJA, GA, IAA, SA, and stress-related elements like light, anaerobic conditions, and low temperature. Similarly, the most abundant category of *cis*-elements in *AhTPP* genes is related to light response. These findings indicate that both *AhTPS* and *AhTPP* genes are involved in distinct hormone regulatory pathways and responses to environmental stresses in cultivated peanut.

### 3.6 Expression analysis of *AhTPS* and *AhTPPs* in various tissues and under different environmental treatments

**Figure 6:**
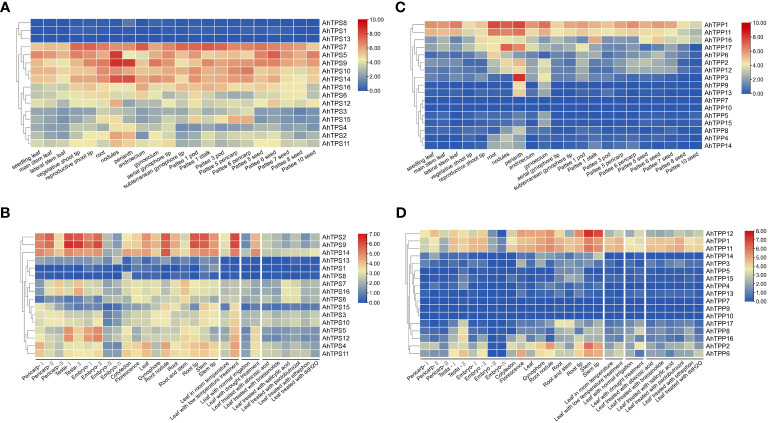
Heat map of *AhTPS* and *AhTPP* genes in different tissues, and under hormones and abiotic stresses. The color scale represents Log_2_ expression values (1+RPKM). **(A)** Expression profiles of *AhTPSs* in 22 different tissues (Clevenger et al., 2016). **(B)** Transcriptome expression of *AhTPSs* under different tissue, hormones and stress conditions (Zhuang et al., 2019). **(C)** Expression profiles of *AhTPPs* in 22 different tissues (Clevenger et al., 2016). **(D)** Transcriptome expression of *AhTPPs* under different tissues, hormones and stress conditions (Zhuang et al., 2019).

The specific expression patterns of genes can indicate their potential roles in growth and development. We utilized publicly available RNA-seq data related to peanut growth and development from the peanut genome database to investigate the expression profiles of 16 *AhTPS* and 17 *AhTPP* genes across various tissues (Clevenger et al., 2016; Zhuang et al., 2019). Furthermore, we conducted a detailed analysis of the expression pattern changes of *AhTPSs* and *AhTPPs* in leaves under different hormonal, low temperature, and drought treatments using a publicly available transcriptome dataset (**Figure 6**). The results suggested that there were differences in the expression levels of the *AhTPS* genes in many tissue types across multiple developmental stages.

In both sets of transcriptomic data, the expression levels of *AhTPS1*, *AhTPS8*, and *AhTPS13* are consistently low across various tissues and treatments, indicating a potential loss of gene function during evolution. Conversely, other *AhTPS* genes exhibit differential expression across different developmental stages and tissues. Certain genes, such as *AhTPS9* and *AhTPS14* (**Figures 6A,B**), maintain consistently high expression levels across nearly all developmental stages and tissues. Some genes, like *AhTPS5* and *AhTPS12*, are predominantly expressed in seed coats and early embryos, while *AhTPS2*, *AhTPS9*, and *AhTPS14* show significantly higher expression levels in roots and root nodules compared to other *AhTPS* genes. Moreover, the expression levels of almost all *AhTPS* genes decline during late embryonic development stages (Embryo-III and Embryo-IV) (**Figure 6B**). These findings suggest diverse functional roles of different *TPS* genes in various tissues and developmental stages. Under drought and low-temperature treatments, *AhTPS* genes exhibit a notable increase in expression, particularly *AhTPS2*, *AhTPS9*, and *AhTPS14*, suggesting their potential importance in peanut’s response to adverse conditions. Regarding hormone treatments, the expression levels of *AhTPS6*, *AhTPS7*, and *AhTPS16* significantly increase in response to salicylic acid and paclobutrazol treatment, while other *AhTPS* genes do not show significant expression level changes in response to hormone treatments.

As for *AhTPP* genes, closely related homologs *AhTPP1* and *AhTPP11* share similar expression patterns and significantly higher expression levels across various tissues compared to other *AhTPP* genes (**Figures 6C, D**). On the other hand, *AhTPP7*, *AhTPP9*, *AhTPP10*, and *AhTPP13* consistently exhibit low expression levels across both transcriptomic datasets. *AhTPP* gene expression also varies in different developmental stages and tissues. For instance, *AhTPP3* show higher expression levels in floral organs (**Figure 6C**), while *AhTPP12* and *AhTPP17* display elevated expression levels in root tissues. Despite some changes in expression levels in response to stress and hormone treatments, *AhTPP* genes do not exhibit the same magnitude of expression differences as observed in *AhTPS* genes (**Figure 6D**).

### 3.7 Real-time expression of *AhTPSs* and *AhTPPs* under cold treatment

**Figure 7:**
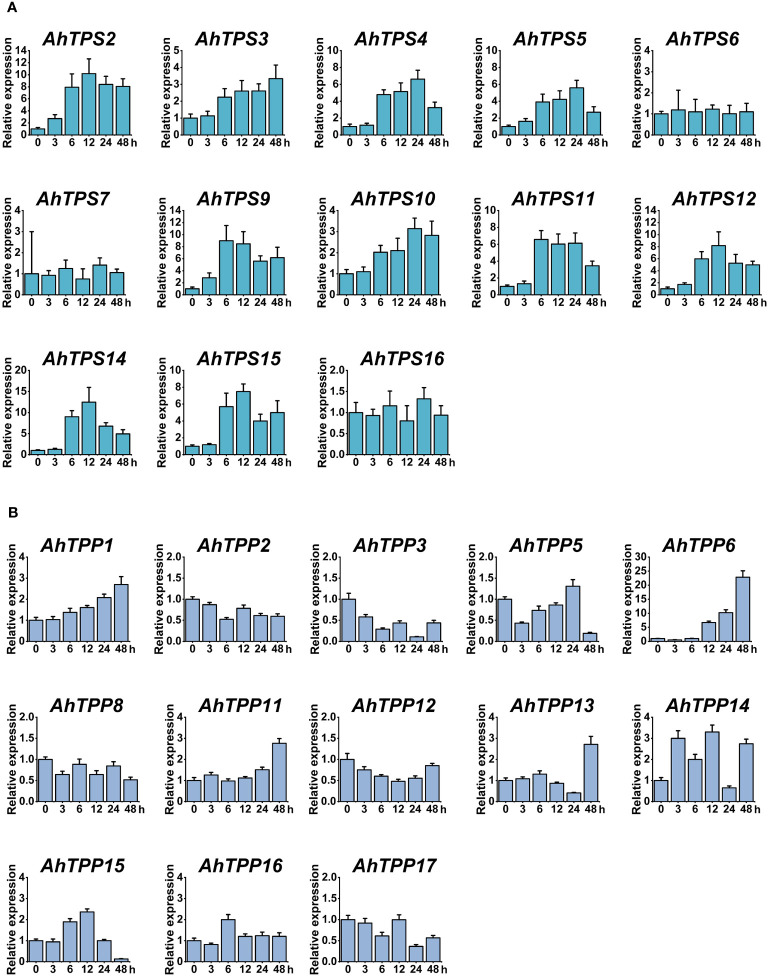
Expression patterns of *AhTPS* **(A)** and *AhTPP* **(B)** genes in leaf tissues under low-temperature stress. Data represents the mean ± standard deviation of three biological replicates each with three technical replicates. Relative transcript levels of the selected genes were calculated using the 2^-ΔΔCt^ method, with *Actin* gene in cultivated peanut as the internal reference.

To further explore the expression patterns of the *AhTPS* and *AhTPP* genes under low-temperature conditions, we employed qPCR to assess the changes in expression levels of these genes at different time points in the leaves (**Figure 7**). Primers used for this experiment are included in **Supplementary Table 9**. Consistent with the expression patterns from transcriptomic data, no expression levels were detected for *AhTPS1*, *AhTPS8*, and *AhTPS13* within the *AhTPS* gene family, as well as *AhTPP4*, *AhTPP7*, *AhTPP9*, and *AhTPP10* within the *AhTPP* gene family at any time point under low-temperature treatment (**Figure 7A**). However, the remaining genes were all detectable. Within the *AhTPS* gene family, *AhTPS2-5*, *AhTPS9-12*, *AhTPS14*, and *AhTPS15* exhibited varying degrees of upregulation in their expression levels under low-temperature treatment. Except for *AhTPS3*, which reached its highest expression level at 48 hours post-treatment, the other *AhTPS* genes reached their expression peaks at 12 or 24 hours, indicating a relatively similar expression pattern for these genes under low-temperature treatment. On the other hand, *AhTPS6*, *AhTPS7*, and *AhTPS16* showed insignificant differences in expression levels under low-temperature treatment, suggesting that these genes might not respond to cold induction. Compared to the *AhTPS* genes, most of the *AhTPP* genes did not exhibit substantial differences in expression levels under low-temperature treatment. *AhTPP1*, *AhTPP6*, *AhTPP11*, and *AhTPP13* showed elevated expression levels under low-temperature treatment, with the highest expression level observed at 48 hours post-treatment (**Figure 7B**). Among these, the most significant alteration in expression levels under low-temperature treatment was observed for *AhTPP6*. However, the expression levels of other genes did not display significant changes.

### 3.8 Subcellular localization of *AhTPS9*

**Figure 8:**
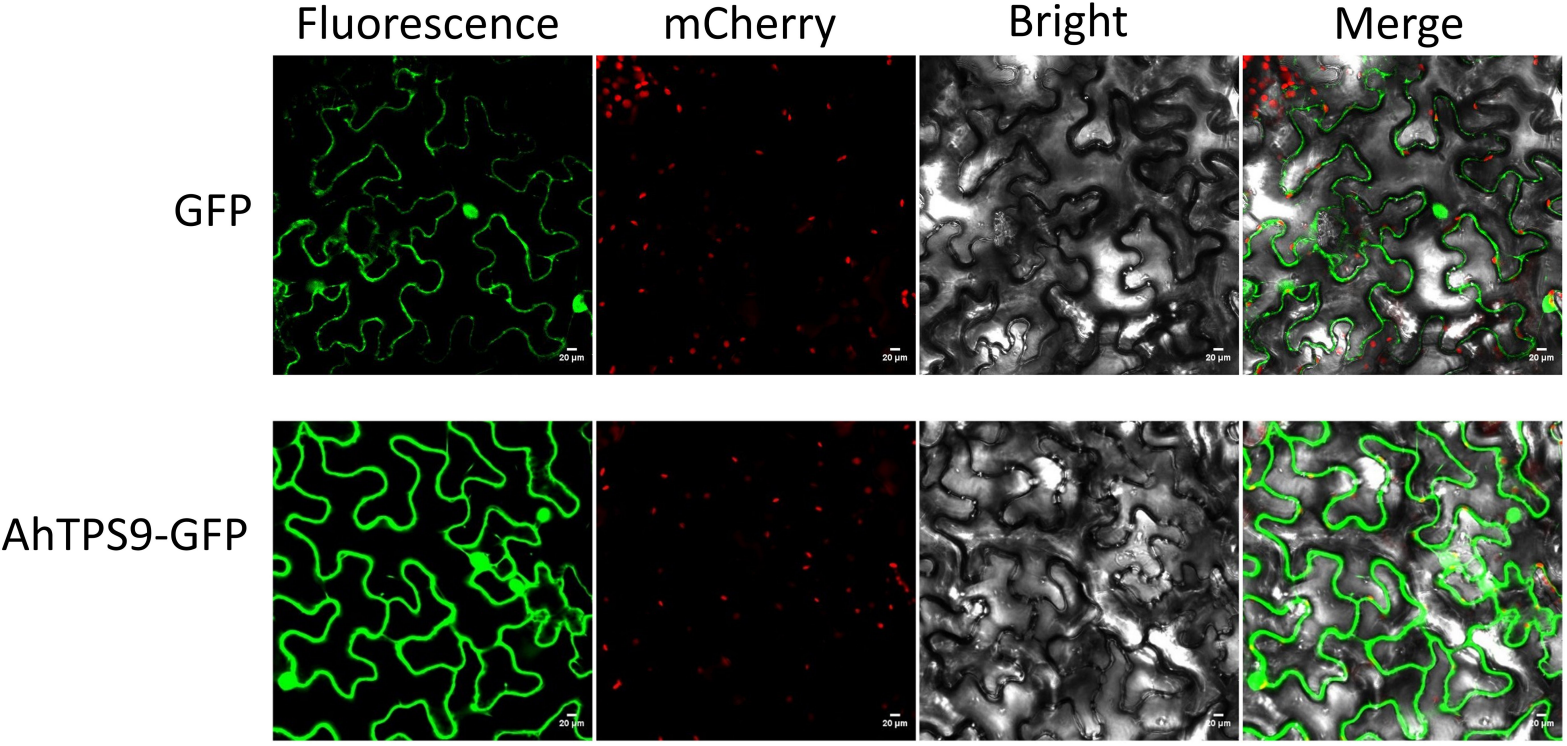
The subcellular localization of *AhTPS9* in *Nicotiana benthamiana* leaf epidermal cells is depicted utilizing a confocal fluorescence microscope. Scale bars represent 20 μm.

In order to further elucidate the protein function and expression patterns of the *AhTPS* gene in peanut, and in conjunction with findings derived from transcriptomic analysis and qPCR validation, the *AhTPS9*, displaying the most pronounced differential expression under low-temperature conditions, was specifically chosen for subcellular localization analysis. The subcellular distribution of the *AhTPS9* protein was investigated using the *Agrobacterium*-mediated transient expression technique, with green fluorescent protein (GFP) tagging, in the epidermal cells of tobacco leaves. Microscopic imaging of fluorescence revealed that, in leaves transformed with the control vector, GFP exhibited a uniform distribution across the epidermal cells (**Figure 8**). Furthermore, subsequent to the transient expression of AhTPS9-GFP fusion proteins in tobacco epidermal cells, the GFP signals were observed within both the cytoplasm and the nucleus. These results collectively indicate the dual localization of AhTPS9 within both the nucleus and cytoplasm of tobacco leaves.

### 3.9 Overexpression of *AhTPS9* confers cold stress in transgenic *Arabidopsis*

**Figure 9:**
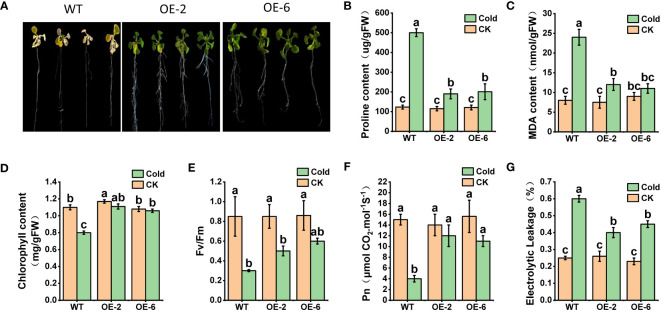
Phenotypes of *Arabidopsis* overexpressing *AhTPS9*, OE2 and OE6, along with the wild-type (WT) control, following a 72-hour cold treatment. Arabidopsis plants were grown on MS medium for 14 days and subsequently exposed to a cold environment at 4°C for 72 hours **(A)**. The endogenous levels of stress-responsive parameters, including Proline **(B)** and MDA **(C)**, were measured. Additionally, photosynthesis-related parameters under cold conditions were assessed, including chlorophyll content **(D)**, Fv/Fm **(E)**, net photosynthetic rate (Pn) **(F)**, and electrical leakage **(G)**. The data presented are expressed as the means ± standard errors (SEs) derived from three independent biological replicates. Statistically significant distinctions among the lines are denoted by different lowercase letters (a–c) based on Duncan’s test (*p*-value < 0.05).

We conducted an analysis of *AhTPS9* function under cold stress conditions in transgenic *Arabidopsis* plants. Two transgenic *Arabidopsis* lines, *AhTPS9-OE2* and *AhTPS9-OE6*, overexpressing *AhTPS9*, were generated through the construction of *AhTPS9* overexpression vectors and genetic transformation of *Arabidopsis*. Since *AhTPS9* is induced by low-temperature stress, both wild-type (WT) and various *AhTPS9* overexpression (OE) seedlings were subjected to cold treatment. No observable phenotypic differences under normal conditions (22°C). However, under cold treatment, wild-type *Arabidopsis* plants exhibited a cold-sensitive phenotype compared to the two *AhTPS9* overexpression lines (**Figure 9**). Physiological indicators indicated lower levels of proline and MDA in the *AhTPS9* overexpression lines under low-temperature treatment, suggesting that *AhTPS9* can mitigate cold-induced damage in plants.

**Figure 10:**
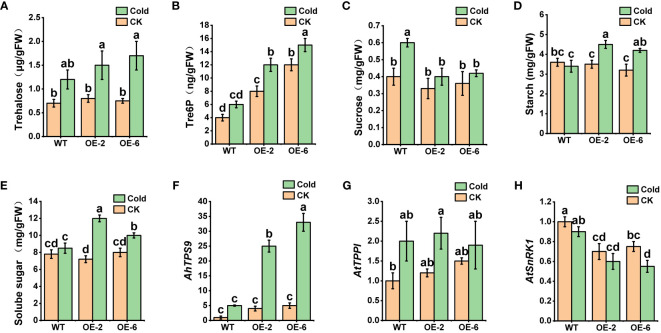
Changes in sugar metabolism and related genes in *AhTPS*9 overexpressing transgenic plants, including **(A)** Trehalose content, **(B)** Tre6P content, **(C)** sucrose content, **(D)** starch content, **(E)** soluble sugar content, as well as the relative expression levels of *AhTPS9*, *AtTPPI*, and *AtSnRK1* (**F–H**). The data are presented as means ± standard errors (SEs) derived from three independent biological replicates. Statistically significant differences among the lines are indicated by different lowercase letters (a–c) based on Duncan’s test (*p*-value < 0.05).

The measurement of key indicators related to the photosynthetic system revealed that, under normal temperature conditions, there were no significant differences between wild-type *Arabidopsis* (WT) and the overexpressing plants (*AhTPS9-OE2* and *AhTPS9-OE6*) in terms of Chlorophyll content, Fv/Fm (maximum quantum yield of photosystem II), Pn (photosynthesis rate), and Electrolytic Leakage. However, under low-temperature treatment, the wild-type plants exhibited a significant reduction in Chlorophyll content, Pn, and Fv/Fm, while showing an increase in electrolytic leakage compared to *AhTPS9* overexpressing plants (*AhTPS9-OE2* and *AhTPS9-OE6*). This indicates that the overexpression of *AhTPS9* has the ability to alleviate the damage caused by low temperatures to the photosynthetic system in *Arabidopsis thaliana*. Further analysis of sugar metabolites and gene expression levels in the trehalose biosynthesis pathway revealed that, under low-temperature conditions, *AhTPS9* expression was significantly higher in the overexpression lines than in the wild type. Additionally, downstream *AtTPPI* gene expression levels were also elevated in the *AhTPS9-OE*2 and *AhTPS9-OE6* compared to the wild type (**Figure 10**). Moreover, the overexpression lines exhibited higher levels of Tre6p and trehalose but lower levels of sucrose compared to the wild type. In contrast, the overexpressing plants of *AhTPS9* exhibited significantly higher levels of soluble sugars and starch content compared to the wild-type plants. The expression levels of key genes related to sucrose and Tre6P, such as *AtSnRK1*, were also examined. It was observed that, under normal temperature conditions, the expression of *AtSnRK1* was higher in the WT plants than in *AhTPS9* overexpressing plants. However, under cold stress conditions, the expression levels of *AtSnRK1* significantly decreased. These findings suggest that *AhTPS9* may regulate sugar metabolism pathways to alleviate the damage caused by low-temperature stress. Overall, our study demonstrates that *AhTPS9* plays a crucial role in enhancing plant tolerance to cold stress, potentially by modulating sugar metabolism pathways.

## 4 Discussion

In response to low-temperature stress, plants rely on molecular mechanisms, with the trehalose biosynthesis pathway and its associated genes playing a crucial role. In plants, trehalose plays a role in regulating the response of plants to various environmental stresses (Paul et al., 2008). Recent research has suggested that the low levels of trehalose in plants may be involved in regulating their response to environmental stress in conjunction with its precursor, Tre6P (Dijken et al., 2004; Schluepmann et al., 2004; O’Hara et al., 2013). This implies that trehalose within plants may serve as an important signaling molecule in mediating the perception and regulation of both biotic and abiotic stresses. Therefore, investigating the trehalose signaling pathway in plants will contribute to the refinement of the plant sugar signaling network and uncovering the mechanisms by which sugars participate in regulating plant growth and development. Key genes in this context include *TPS* and *TPP* genes (Eastmond and Graham, 2003). These gene families regulate the synthesis and metabolism of trehalose, serving as vital components in the plant’s response to low temperatures (Kandror et al., 2002; Iordachescu and Imai, 2008). Numerous studies have been conducted on various plant species, such as *Arabidopsis*, rice, and common bean, revealing the expression and functions of *TPS* and *TPP* genes in cold stress responses (Fernandez et al., 2010; Liu et al., 2019; Lin et al., 2023; Liu et al., 2023). They contribute to the maintenance of membrane stability, protein protection, and ultimately enhance the plant’s cold resistance (Elbein et al., 2003). Therefore, in this study, we identified the *TPS* and *TPP* genes involved in the synthesis pathway of trehalose in peanuts and explored their roles in the response to low temperatures.

In this study, a total of 16 *AhTPS* and 17 *AhTPP* genes were identified in the cultivated peanut genome, while in the genome of wild diploid peanut *A. duranensis*, there were 9 *AdTPS* genes and 7 *AdTPP* genes, and in the genome of *A. ipaensis*, there were 10 *AiTPS* genes and 8 *AiTPP* genes (**Figure 1**, **Table 1**). Most of the *TPS* and *TPP* gene family members in wild diploid peanuts showed orthologous relationships with genes in the A and B subgenomes of cultivated peanuts, with differences in their numbers possibly arising from new duplication events between the two subgenomes of cultivated peanuts. Although cultivated peanuts have a larger number of *AhTPS* and *AhTPP* genes compared to other species such as *Arabidopsis*, rice, soybean, tomato, watermelon and cucumber (Vogel et al., 2001; Zang et al., 2011; Dan et al., 2021; Yuan et al., 2021; Lin et al., 2023), this may be attributed to the fact that cultivated peanuts, being tetraploids derived from two wild diploid peanuts, *A. duranensis* and *A. ipaensis*, have larger genomes (Bertioli et al., 2016; Bertioli et al., 2019; Zhuang et al., 2019). The *AhTPS* and *AhTPP* genes have been classified into two main groups based on other crops such as *Arabidopsis*, rice, and soybean (**Figure 6**). The *AhTPS* and *AhTPP* gene sequences are relatively conserved, with *TPS* or *TPP* orthologous genes within the *Arachis* species clustering together. However, the similarity between non-orthologous genes within the *Arachis* species is lower than the relationship between the *Arachis* species and the leguminous crop soybean. This implies that *AhTPS* genes and *AhTPP* genes exhibit a higher degree of conservation across species, while the similarity among family members is lower than that between species. Different *AhTPS* and *AhTPP* genes may undergo functional differentiation, and various *AhTPS* and *AhTPP* members may share similar functions with soybean. Gene duplication plays a crucial role in evolutionary processes, including chromosomal rearrangements, the diversification of gene functions, and the enlargement of gene families (Cannon et al., 2004; Huang and Rieseberg, 2020; Mérot et al., 2020). Hence, the duplication events of *AhTPS* and *AhTPP* gene members during the evolutionary process were identified, and the results indicate that both gene families, *AhTPS* and *AhTPP*, exhibit similarities in their evolutionary history. This is manifested by the absence of tandem duplications in both gene families, with evolution primarily relying on segment duplications, both in wild diploid peanuts and cultivated tetraploid peanuts (**Figure 3**). Therefore, the combination of phylogenetic and collinearity analysis results demonstrates that the *AhTPS* and *AhTPP* genes maintain a relatively conserved orthologous relationship among species, and the evolutionary process within the peanut species predominantly involves segment duplication.

The analysis of promoter *cis*-acting elements and gene expression patterns reveals that members of the *AhTPS* and *AhTPP* gene families play crucial roles in the growth, development, hormonal regulation, and stress responses of cultivated peanuts (**Figures 5**–**7**). The regulation pathways of trehalose in plant growth, development, and stress responses are intricate. Trehalose plays a vital role in safeguarding bioactive compounds and cellular components, such as proteins, nucleic acids, and biological membranes, from harsh environmental conditions like high salinity, drought, extreme temperatures, freezing, and oxidative stress (Nunes et al., 2013; O’Hara et al., 2013). Its most crucial role lies in modulating the synthesis and metabolism of carbohydrates in response to various biological processes and stressors (Oszvald et al., 2018; Hassan et al., 2023). In this study, both the *AhTPS* and *AhTPP* gene families were found to contain a significant number of light-responsive elements (**Figure 5**), suggesting their potential importance in photosynthesis and energy-related pathways. Combining data from various transcriptome databases and qPCR validation (Bertioli et al., 2019; Zhuang et al., 2019), some of these genes exhibited expression throughout the entire growth process of peanuts and participated in different stress and hormone regulatory mechanisms. Notably, the *AhTPS* family members, *AhTPS2*, *AhTPS9*, and *AhTPS14*, as well as the *AhTPP* family members, *AhTPP1* and *AhTPP11*, were identified to be involved in such processes. Additionally, *AhTPS2* and *AhTPS9*, along with *AhTPP1* and *AhTPP11*, represented two pairs of orthologous genes originating from the A and B subgenomes, as depicted in **Figure 2**. These orthologous genes have been retained during the evolutionary process of wild diploid peanut species (**Figure 1**), indicating their potential significance within the *Arachis* genus.

To our knowledge, the manipulation of the *Arachis hypogaea AhTPS* genes in plants has not previously been reported. Combining bioinformatics and gene expression analysis, we observed that the expression levels of *AhTPS2* and *AhTPS9* were significantly higher than others (**Figure 6**, **Figure 7**). These genes exhibited similar gene structures and regulatory elements and showed similar expression levels in different tissues, hormonal treatments, and stress conditions. This suggests that they may have similar functions in the cultivated peanut genome, potentially indicating functional redundancy. To further explore the role of *AhTPS* genes under cold stress, *AhTPS9* was selected for heterologous transformation into *Arabidopsis* plants to validate its function. Regulation of *TPS* genes has been shown to enhance abiotic stress tolerance in plants (Pilon-Smits et al., 1998; Garg et al., 2002; Chary et al., 2008; Fernandez et al., 2010; Li et al., 2011; Wang et al., 2016).For instance, constitutive expression of yeast *ScTPS1* in potatoes improved drought tolerance but led to pleiotropic growth abnormalities, such as dwarfism, chlorotic leaves, and aberrant root development (Yeo et al., 2000). Transgenic tomato expressing ScTPS1 exhibited improved drought and salt tolerance but also showed other phenotypic changes (Cortina and Culiáñez-Macià, 2005). In tobacco, *ScTPS1* expression increased drought tolerance without growth aberrations (Karim et al., 2007). Similarly, overexpression of *OsTPS1* in rice improved tolerance to cold, salinity, and drought without visible phenotypic changes (Li et al., 2011). Overexpression of *Triticum aestivum TaTPS11* in *Arabidopsis* enhanced cold tolerance without adverse phenotypes, suggesting its potential value in wheat cold-tolerance breeding (Liu et al., 2019). In this study, in *Arabidopsis* plants overexpressing *AhTPS9*, compared to wild-type plants, no significant phenotypic changes were observed during germination and growth processes. However, under cold stress, the AhTPS9-overexpressing plants exhibited improved tolerance, showing reduced levels of proline and MDA, and without significant wilting. This suggests that *AhTPS9*, similar to *OsTPS1* and *TaTPS11*, can alleviate stress without affecting key phenotypic changes. *AhTPS9* can mitigate the damage to the photosynthetic system caused by low temperatures, including chlorophyll content, Pn, and Fv/Fm, which were significantly higher in the overexpressing plants compared to the wild type. *TPS* genes and their product, Tre6P, have been reported to enhance photosynthesis under stress conditions (Paul et al., 2001). In maize, Tre6P has been shown to regulate photosynthesis and assimilate distribution in reproductive tissues (Oszvald et al., 2018). In tomatoes, co-expression of *TPS* and *TPP* enhances photosynthesis under drought and salt stress without affecting plant growth (Lyu et al., 2013). *AhTPS9* contains abundant photosynthesis-related elements (**Figure 5**) and is regulated by multiple hormones (**Figure 6**). Therefore, the specific regulatory mechanism of *AhTPS9* in protecting photosynthesis under low-temperature conditions requires further research.

Sugar metabolism plays a crucial role in plant responses to low-temperature stress (Nägele and Heyer, 2013; Kovi et al., 2016; Hassan et al., 2023). As a soluble sugar, trehalose is present at extremely low levels, making it unable to provide the necessary osmotic stress protection independently. However, the intermediate product Tre6P plays a critical role in sucrose regulation. Tre6P acts as both a signal for sucrose levels and a negative feedback regulatory factor, contributing to the maintenance of sucrose levels within an appropriate range (Figueroa and Lunn, 2016). Tre6P can interact with *Sucrose Non-Fermenting 1-Related Kinase 1* (*SnRK1*) to regulate sucrose levels antagonistically (Baena-Gonzalez and Lunn, 2020). In this study, we also examined key genes related to trehalose metabolism and their metabolic products in transgenic Arabidopsis and its wild type. The results showed that AhTPS9-overexpressing plants exhibited significantly higher levels under low-temperature conditions, leading to the accumulation of Tre6P and a decrease in sucrose levels, which is consistent with the model of Tre6P-SnRK1 regulation of sucrose levels proposed by previous studies (Baena-Gonzalez and Lunn, 2020). *AtTPP1* can enhance *Arabidopsis* cold tolerance by accumulating soluble sugars and jasmonic acid. In this study, the expression level of this gene increased under low-temperature treatment, but it was not affected by *AhTPS9*. However, it resulted in higher levels of trehalose, suggesting that the upregulation of this gene’s expression may be regulated by CBF genes (Lin et al., 2023). Overexpression of *AtTPS11* in *Arabidopsis* under low-temperature conditions significantly increased starch content (Singh et al., 2011). As an orthologous gene (**Figure 1**), *AhTPS9* similarly increased starch content in overexpressing plants under low-temperature conditions. However, soluble sugar accumulation in overexpressing plants was significantly higher than in the wild type under low-temperature conditions. Therefore, there are complex interactions between sugar metabolism-related genes in the trehalose pathway and cold tolerance, which require further detailed investigation.

## 5 Conclusion

Collectively, the current study identified 16 *AhTP*S and 17 *AhTPP* genes in the peanut genome through bioinformatics analysis. Phylogenetic analysis revealed two distinct subgroups closely related to wild diploid peanuts. Evolutionary patterns suggested gene segmental duplication events and robust purifying selection. Based on expression pattern analysis, *AhTPS9* exhibits the most significant differential expression under cold stress. Functional validation revealed that *Arabidopsis* plants overexpressing *AhTPS9* demonstrate enhanced cold tolerance by improving the photosynthetic system and regulating related products and genes involved in sugar metabolism.

## Data availability statement

The original contributions presented in the study are included in the article/**Supplementary Material**. Further inquiries can be directed to the corresponding author.

## Author contributions

CZ: Conceptualization, Data curation, Formal analysis, Funding acquisition, Investigation, Methodology, Writing – original draft, Writing – review & editing. ZH: Conceptualization, Data curation, Formal analysis, Investigation, Methodology, Writing – original draft, Writing – review & editing. YL: Data curation, Formal analysis, Investigation, Writing – review & editing. ZL: Data curation, Formal analysis, Investigation, Writing – review & editing. XW: Data curation, Formal analysis, Project administration, Supervision, Validation, Visualization, Writing – review & editing. CJ: Data curation, Formal analysis, Project administration, Supervision, Validation, Visualization, Writing – review & editing. SK: Data curation, Formal analysis, Project administration, Supervision, Validation, Visualization, Writing – review & editing. XL: Data curation, Formal analysis, Project administration, Supervision, Validation, Visualization, Writing – review & editing. SZ: Data curation, Formal analysis, Project administration, Supervision, Validation, Visualization, Writing – review & editing. JW: Data curation, Formal analysis, Project administration, Supervision, Validation, Visualization, Writing – review & editing. HZ: Data curation, Formal analysis, Project administration, Supervision, Validation, Visualization, Writing – review & editing. XZ: Data curation, Formal analysis, Project administration, Supervision, Validation, Visualization, Writing – review & editing. HY: Funding acquisition, Resources, Supervision, Validation, Visualization, Writing – review & editing.
