# Supplementary figures and images for: Genome-wide identification of TPS and TPP genes in cultivated peanut (Arachis hypogaea) and functional characterization of AhTPS9 in response to cold stress
Source: Front Plant Sci. 2024 Jan 19;14:1343402. doi: 10.3389/fpls.2023.1343402 (PMC10834750; doi:10.3389/fpls.2023.1343402)

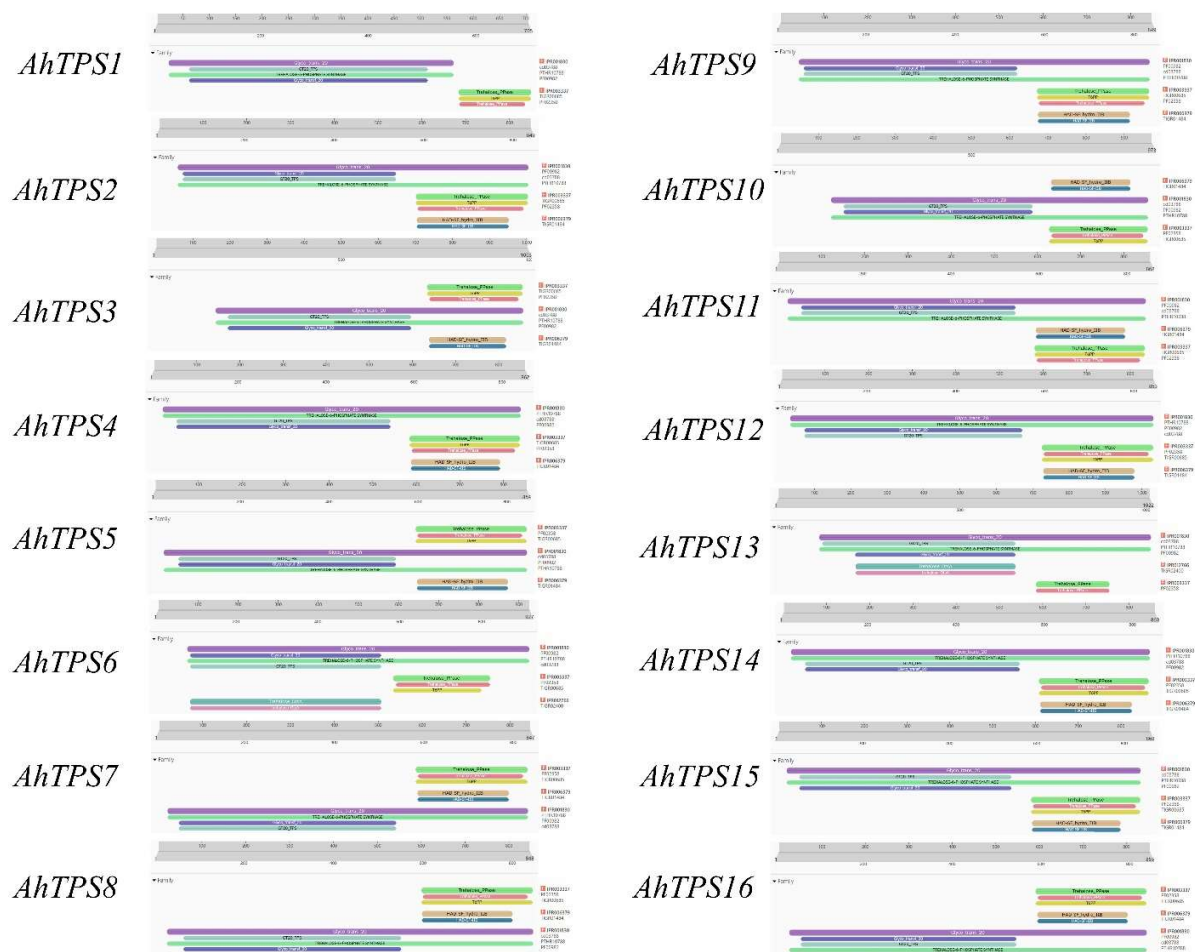

**Figure S1.** Conserved domains of *AhTPS*s.

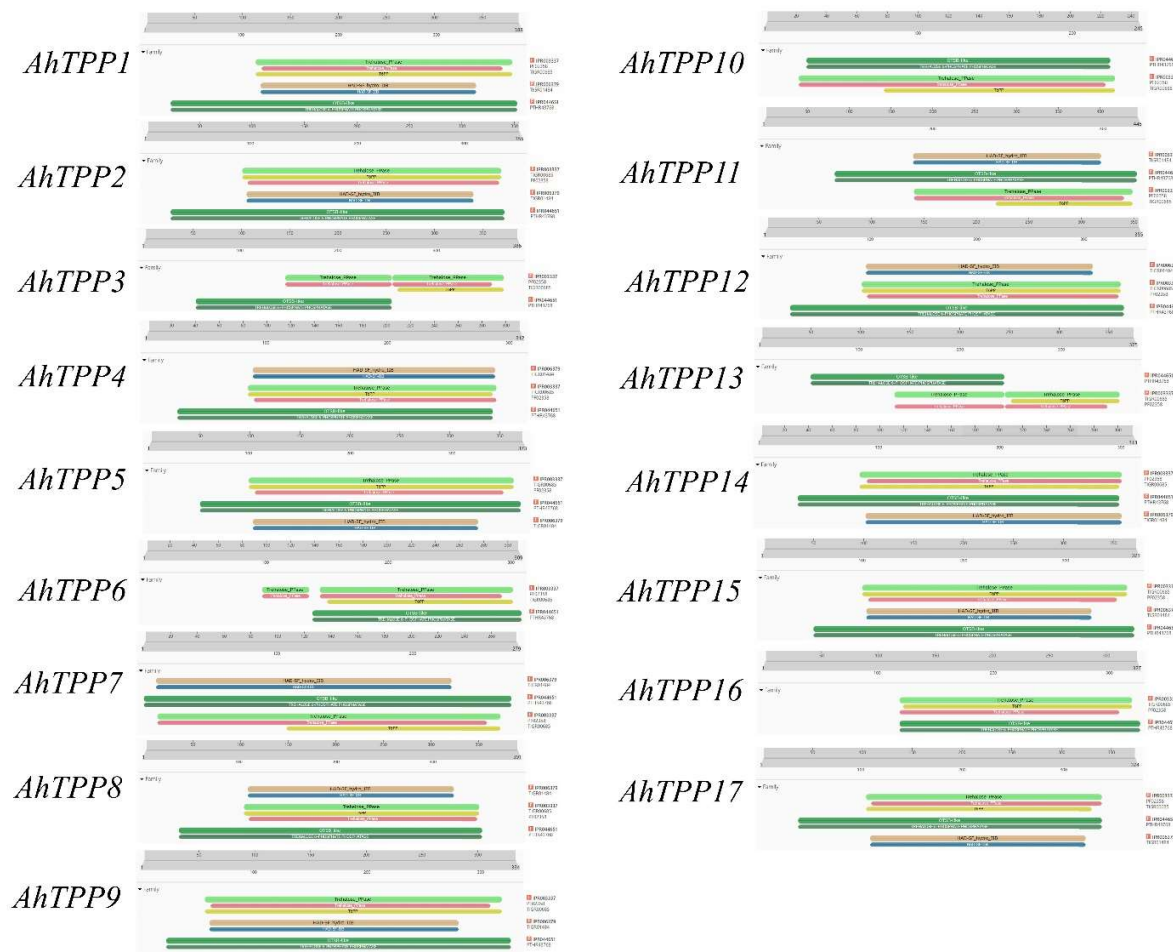

**Figure S2.** Conserved domains of *AhTPPs*.

Supplement: Supplementary file 1 [file DataSheet_1.zip › Figure S1 S2.pdf]
